# Differences according to Sex in Sociosexuality and Infidelity after Traumatic Brain Injury

**DOI:** 10.1155/2015/914134

**Published:** 2015-10-12

**Authors:** Jhon Alexander Moreno, Michelle McKerral

**Affiliations:** ^1^Center for Interdisciplinary Research in Rehabilitation (CRIR), Centre de Réadaptation Lucie-Bruneau (CRLB), 2275 Laurier Avenue East, Montréal, QC, Canada H2H 2N8; ^2^Centre de Recherche en Neuropsychologie et Cognition (CERNEC), Département de Psychologie, Université de Montréal, Montréal, QC, Canada

## Abstract

*Objective*. To explore differences according to sex in sociosexuality and infidelity in individuals with TBI and in healthy controls. *Participants*. Forty-two individuals with mild, moderate, and severe TBI having completed a postacute TBI rehabilitation program, at least six months after injury, and 47 healthy controls. *Main Measures*. Sociosexual Orientation Inventory-Revised (SOI-R) and Attitudes toward Infidelity Scale. *Results*. Overall, men score significantly higher than women in sociosexuality. However, there was a nonsignificant trend towards a reduction of sociosexuality levels in men with TBI. Infidelity levels were comparable in healthy controls and individuals with TBI. In individuals with TBI, less acceptance of infidelity was significantly associated with an unrestricted sociosexual orientation, but not in healthy controls. *Conclusions*. As documented in previous cross-cultural studies, men have higher levels of sociosexuality than women. However, men with TBI showed a tendency towards the reduction of sociosexuality. The possibility of a latent explanatory variable is suggested (e.g., post-TBI neuroendocrinological changes). TBI does not seem to have an impact on infidelity, but individuals with TBI who express less acceptance of infidelity also report a more promiscuous mating strategy regarding their behavior, attitudes, and desire. Theoretical implications are discussed in terms of evolutionary theories of human sexuality and neuropsychology.

## 1. Introduction

Nonmonogamy is part of the evolutionary trends preserved in humans [1]. In fact, infidelity constitutes probably one of the most complex problems faced by mental health professionals, especially couple therapists, marriage and family therapists, and psychotherapists [2, 3]. Based on evolutionary theories, there are sex differences regarding reactions to infidelity. For instance, men seem to be more distressed by sexual infidelity (e.g., sexual relationship or sexually oriented physical contact with another person), while women may be more distressed by emotional infidelity (e.g., diversion of the partner's emotional commitment toward another person) [4]. Interestingly, human brains show different activation patterns in response to different types of infidelity; men and women process sexual and emotional infidelity using different neuropsychological networks [5].

Nonetheless, the experience of infidelity is linked to the individuals' proneness to be unfaithful. This is the research area of sociosexuality, also known as sociosexual orientation (SO). Kinsey was the first to introduce the term in his pioneer studies describing individual differences in people's willingness to engage in uncommitted sexual relationships [6, 7]. Sociosexuality levels range from an unrestricted SO to a restricted SO. Individuals with an unrestricted SO tend to a more promiscuous mating strategy, are quicker to have sex, and may experience lower levels of romantic relationship closeness or commitment. Conversely, individuals with a restricted SO tend to a more monogamous mating approach, invest more time in courtship, and develop strong emotional connections in long-term relationships [8].

Undoubtedly, the most striking cross-cultural evidence of the existence of SO comes from the international sexuality description project [9]. This groundbreaking analysis of sociosexuality in 48 nations demonstrated that, compared to women, men have higher levels of sociosexuality across cultures and that sex differences in sociosexuality are culturally universal. Furthermore, even though sex differences in sociosexuality are attenuated in cultures with more gender equality in terms of political, economic, and relational freedom, the findings of this study did not suggest that men and women tend to become equally promiscuous in attitudes and behaviors. Research has demonstrated the existence of a sociosexuality-testosterone association in both men and women and revealed that the nature of these associations varies by gender and relationship status (e.g., partnered men who reported an unrestricted sociosexuality had testosterone levels that were comparable to those of single men) [10]. However, research in the area of sociosexuality has included not only differences according to sex [11] but also many other variables, such as racial differences [12], infidelity [13], attachment style [4], self-image [14], physical attractiveness and sexual aggression perpetration [15], and personality styles [16], among others.

Overall, the aforementioned studies highlight the importance of sociosexuality, from both an evolutionary and an environmental/sociocultural perspective to explain the reasons behind the fact that, on average, men are more willing than women to engage in casual sex. Two different interpretations have been suggested. In his seminal theory of parental investment and sexual selection, Trivers (1973) defined parental investment as the resources that a parent spends on his offspring in order to increase the chances of surviving and reproducing, at the cost of this parent's ability to invest in other offspring [17–19]. Together, these studies outline that, from an evolutionary perspective, men have more to gain and less to lose by having sex outside a committed relationship. In contrast, women have to invest time and energy devoted to pregnancy and childbearing. This interpretation contrasts with an environmental/sociocultural perspective, suggesting the possibility that differences in sociosexuality can be associated, in part, with the variations in the regional prevalence of infectious diseases. People in regions with a history of a high prevalence of infectious diseases report lower levels of sociosexuality [20].

Given the evolutionary, neuropsychological, and environmental/sociocultural rationales put forward in the research literature on sociosexuality, its presentation in acquired medical conditions where changes in brain functions are induced warrants investigation. Traumatic brain injury (TBI), which is among the most common neurological conditions [21], is a form of brain injury which is receiving increasing attention in the area of research on sexuality, given its biopsychosocial consequences [22–27].

Also, TBI impacts people's sexuality, with 50 to 60% of persons reporting some level of disruption after injury [28–30], and sexual function is compromised as a result of the post-TBI changes involving the neurological aspects of sexuality [27]. Sexual difficulties after TBI have thus been associated with medical and physical issues (e.g., neuroendocrine and hormonal disorders [31, 32], neuropsychological and psychological effects (e.g., depression [33]), and relationship changes (e.g., intimacy [34])) [25].

To our knowledge, previous studies on sexuality and TBI have not addressed attitudes towards infidelity and sociosexuality in individuals with TBI. The current study thus aimed to explore differences according to sex in sociosexuality and attitudes towards infidelity in individuals with TBI and in healthy controls. In the current study, infidelity is defined as a person being unfaithful while in a committed monogamous relationship. Since this is a novel and exploratory study, no specific hypotheses related to TBI participants are advanced, but it is postulated that there are statistically significant differences according to sex in sociosexuality for healthy controls, with men having higher levels of SO compared to women.

## 2. Methods

### 2.1. Participants

The sample consisted of 42 individuals with TBI and 47 healthy controls. Individuals with TBI were recruited from a TBI outpatient rehabilitation center in Montreal, which offers social and vocational rehabilitation services to individuals with moderate or severe TBI, as well as to individuals with mild or complex mild TBI showing atypical recovery to which the brain injury appears to contribute predominantly. Individuals with TBI were recruited based on the following inclusion criteria: (1) individuals who have sustained, according to the TBI guidelines put forward by the Québec Ministry of Health [35], a mild (Glasgow coma scale (GCS) scores 13–15), moderate (GCS scores 9–12), or severe (GCS scores 3–8) TBI, (2) individuals who are six or more months post-injury, (3) individuals who are 18 years or older, and (4) individuals who report to be able to read, write, and speak either French or English. Exclusion criteria, as verified in medical records, included (1) history of learning or language disability, including aphasia or communication disorders and (2) self-report of preinjury psychiatric, sexual, or neurological disorders other than TBI. A detailed description of the sociodemographic characteristics of the sample is provided in Table 1.

In terms of clinical characteristics, as indicated in Table 2, the majority corresponds to mild TBIs (66.8%). The cause of the injury was predominantly associated with a motor vehicle accident (42.9%) followed by work and sports-related accidents (14.3%). Half of them had a history of loss of consciousness (50%) and 47.6% had also a history of posttraumatic amnesia documented in the medical chart. Individuals with TBI were on average 3.3 years after the injury (SD = 4.3). Positive findings on CT scan or MRI suggesting a brain injury were documented in 59.5%. Glasgow coma scale at admission was on average 12.5 (SD = 3.6), with a loss of consciousness of a mean of 5.8 hours (SD = 28.8) and posttraumatic amnesia duration of 80.8 hours (SD = 203.8) as indicated in medical records.

Healthy controls were recruited from the community following these inclusion criteria: (1) being 18 years or older and (2) reporting to be able to read, write, and speak either French or English. Exclusion criteria included (1) self-reported history of learning or language disability and (2) self-report of diagnosed psychiatric, sexual, or neurological disorders. Their sociodemographic characteristics are presented in Table 1.

### 2.2. Procedure

The current study was approved by the Research Ethics Board (REB) of the Center for Interdisciplinary Research in Rehabilitation of Greater Montreal (CRIR). Data collection was undertaken between April 2013 and August 2014.

From the rehabilitation center's database, a total of 345 individuals with TBI were eligible for participation. Following telephone contact by a person independent of the research project (e.g., archives technician) who proposed participation in the study, 13 of them refused to participate and 224 could not be reached. Individuals with TBI who accepted to participate were mailed two envelopes: (a) a consent form (which included a thorough explanation of the study) and (b) a package containing the questionnaires. Each of the envelopes contained a stamped and addressed envelope so that the participant could return each document independently. Questionnaires and consent forms were sent to 108 individuals with TBI and 42 of them successfully completed and returned both (41 in French and 1 in English).

In the context of a larger sexuality study, healthy controls were recruited from the general community through newspaper advertisements, as well as notices in community centers, universities, and libraries. A total of 242 people from the community expressed their interest to participate in the sexuality study. Following a phone call by the research team to verify inclusion/exclusion criteria, questionnaires and consent forms were sent to 191 healthy controls. Twenty-eight of them did not return both the questionnaires and consent forms while 163 returned them. For the purposes of this study, 47 healthy controls (41 in French and 6 in English) were matched to TBI participants from the database of the aforementioned large sexuality study, based on sociodemographic variables (e.g., age, gender, years of education, annual income, work, and relationship status). Questionnaire data were subsequently analyzed.

Voicemail and email accounts were created in order to receive and answer any questions for individuals with TBI or healthy controls. All participants received a financial compensation of CAN$15 (fifteen Canadian dollars) for their participation after returning their questionnaires and consent forms.

### 2.3. Instruments

#### 2.3.1. Medical History and Demographic Information

Participants completed an in-house short medical and sociodemographic questionnaire that included questions related to participant's age (e.g., number of years), race/ethnicity (e.g., white, Hispanic), gender (e.g., male, female), years of education (e.g., number of years), relationship status (e.g., single, married), annual income (in Canadian dollars), work status (e.g., full time, unemployed), frequency of alcohol (e.g., never to everyday), and recreational drug use (e.g., yes, no). For TBI participants, data regarding preinjury and injury related variables (e.g., severity of injury, number of years after injury, length of loss of consciousness in hours, length of posttraumatic amnesia in hours, and presence/absence of neuroradiological abnormalities) were extracted from medical records.

Each of the participants was administered the following questionnaires.

*Sociosexual Orientation Inventory-Revised (SOI-R)*. The SOI-R is a 9-item self-report questionnaire, each with a 9-point response scale, developed to measure individual differences in willingness to engage in casual, uncommitted sexual relationships [8]. In particular, the SOI-R assesses individual's past behavior in terms of number of casual and changing sex partners, the explicit attitude towards uncommitted sex, and sexual desire for people with whom no romantic relationship exists [36]. Scores for behavior, attitude, and desire facets as well as a total score are obtained [37]. Higher scores on the SOI-R correspond to individuals who have an unrestricted sociosexual orientation (or have a more promiscuous mating strategy) whereas lower scores correspond to restricted sociosexual orientation (or individuals who follow a more monogamous mating strategy). The SOI-R proposes adequate reliability and validity both within and across the diverse range of human cultures [9] and has been used widely in a variety of research and clinical samples [37–44]. For items 1 to 3, values of 1 to 9 should be assigned to the responses. Thus, all nine items have values from 1 to 9 (9-point scale). Item 6 should be reverse-keyed. Items 1 to 3 are aggregated (summed or averaged) to form the behavior facet, items 4 to 6 form the attitude facet, and items 7 to 9 form the desire facet. Finally, all nine items can be aggregated to form a full-scale score that represents the global SO. In the current study, the internal consistency of SOI-R (Cronbach's *α* = 0.89), as well as all of the three facets of the SOI-R, was very good (behavior Cronbach's *α* = 0.91, attitude Cronbach's *α* = 0.84, and desire Cronbach's *α* = 0.88).

*Attitudes toward Infidelity Scale*. This is a 12-item self-report questionnaire to measure the acceptance of infidelity. In the context of this scale, infidelity is defined as a person being unfaithful in a committed monogamous relationship. Each item is rated on a 7-point Likert scale with 1 reflecting the least acceptance of infidelity and 7 the greatest acceptance of infidelity. The lower the total score (12 is the lowest possible score), the less the person's acceptance of infidelity, whereas the higher the total score (84 is the highest possible score), the greater the respondent's acceptance of infidelity [45]. A score of 48 places the person at the midpoint between being very disapproving of infidelity and very accepting of infidelity. Before adding the numbers, score items 2, 5, 6, 7, 8, and 12 must be reversed (e.g., 1 = 7, 2 = 6, 3 = 5, 4 = 4, 5 = 3, 6 = 2, and 7 = 1). After making these changes, the numbers must be added to obtain the full-scale score [46]. A translation/back-translation procedure was implemented in order to obtain the French version that was used in the present study and its internal consistency was good (Cronbach's *α* = 0.79).

### 2.4. Statistical Analyses

Demographic characteristics of individuals with TBI were compared to those of healthy controls using* t*-tests for continuous variables and *χ* ^2^ tests for nominal variables, taking into account a significance level *p* < 0.05.

Two-way between-groups analyses of variance (two-way ANOVA) were performed to explore the impact of sex (e.g., male and female) and group (e.g., individuals with TBI and healthy controls) on sociosexuality.

An independent-samples* t*-test was performed to compare infidelity levels between individuals with TBI and healthy controls. Pearson correlation analyses were used to examine the relationship between sociosexuality facets (behavior, attitude, and desire), infidelity levels, and injury characteristics (years after injury, GCS score, and hours of posttraumatic amnesia) in individuals with TBI.

Statistical analyses were conducted with IBM SPSS version 21 [47].

## 3. Results

Comparison of the sociodemographic characteristics of the TBI and healthy control groups, described in Table 1, indicates that there were no significant differences between groups in terms of age, gender, race/ethnicity, work status, relationship status, years of education, and annual income. Also, both groups were comparable in frequency of alcohol consumption, recreational drug use, and the use of one prescribed medication. Comparison of the sociodemographic and clinical characteristics of the TBI group by gender indicates that there were no significant differences between men and women with TBI in terms of age, race/ethnicity, work status, relationship status, years of education, annual income, alcohol consumption, recreational drug use, medication intake, injury severity, time after injury, neuroimaging evidence of brain injury, or loss of consciousness/posttraumatic amnesia duration (all *p*'s > 0.05).

**Figure 1:**
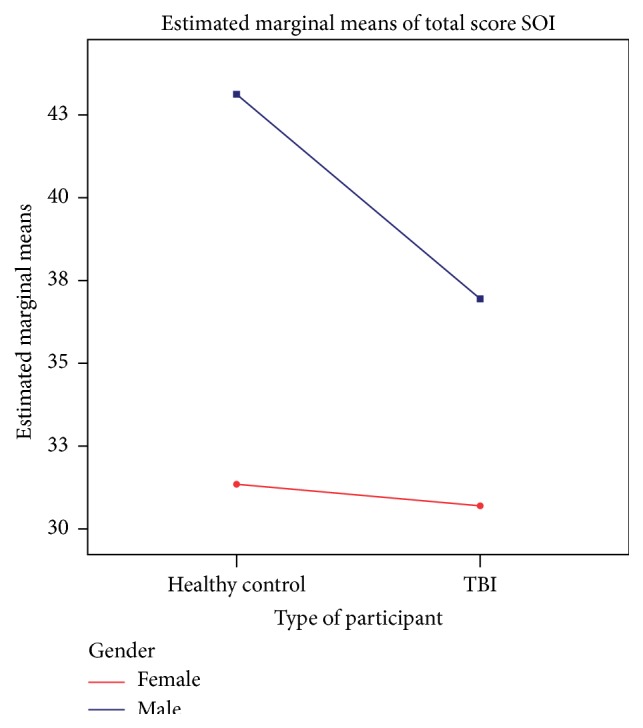
Estimated marginal means for sociosexuality as a function of group and gender. Abbreviation: SOI, total score of the Sociosexual Orientation Inventory.

As summarized in Table 3, a two-way between-groups analysis of variance was performed to explore the impact of sex (male-female) and group (individuals with TBI and healthy controls) on sociosexuality, as measured by the SOI-R. The interaction effect between sex and group was not statistically significant, *F*(1,85) = 0.6, *p* > 0.05. There was a statistically significant main effect for sex, *F*(1,85) = 7.2, *p* < 0.05; and the effect size was in the range of medium to large effect size (partial eta squared = 0.07) according to the guidelines for the behavioral sciences [48]. The main effect for group, *F*(1,85) = 1.0, *p* > 0.05, did not reach statistical significance. Compared to females, overall, males had higher levels of sociosexuality. However, there appeared to be a tendency towards a reduction of sociosexuality levels in males with TBI (see Figure 1).

Finally, compared to healthy controls, individuals with TBI did not show statistically significant differences in infidelity, as measured by the total score of the Attitudes toward Infidelity Scale, *t*(85) = −0.8, *p* > 0.05.

### 3.1. Correlation Matrix

The relationship between infidelity (as measured by the Attitudes toward Infidelity Scale), sociosexuality (as measured by the SOI-R), and TBI characteristics (severity as measured by the score on the GCS scale and by length of posttraumatic amnesia in hours, years after injury) in the group of individuals with TBI was investigated using Pearson product-moment correlation coefficient (see Table 4). There was a large negative correlation between the scores on the infidelity scale and the SOI-R (*r* = −0.58, *p* < 0.01), with low levels of infidelity scores (less permissiveness regarding infidelity) associated with high levels of SO (unrestricted SO). In addition, infidelity scores were moderately associated with behavioral sociosexuality (*r* = −0.34, *p* < 0.05) and sociosexual desire (*r* = −0.49, *p* < 0.01). Also, infidelity scores showed a large correlation with sociosexual attitudes (*r* = −0.57, *p* < 0.01). In contrast, these associations were not significant in the group of healthy controls (all* p*'s > 0.05).

Finally, neither infidelity scores nor sociosexuality was associated with severity of the injury (GCS score or length of posttraumatic amnesia), or with time since injury (all *p*'s > 0.05).

## 4. Discussion

The current study aimed to explore differences according to sex in sociosexuality and attitudes towards infidelity in individuals with TBI and healthy controls. The main finding of the current study is that, compared to healthy controls, our TBI sample appeared to show a tendency towards a reduction of differences according to sex in sociosexuality. Interestingly, there was a trend suggesting a decrease in sociosexuality levels in men with TBI. To our knowledge, this study is the first suggesting the possibility of a decline of this cross-cultural and evolutionary distinction following TBI in males. This finding is important since it could suggest that a complex and deeply rooted psychosexual trait, such as sociosexuality, could be modified after a neurological insult such as TBI.

The tendency towards the reduction of differences according to sex in sociosexuality levels following TBI does not seem to be explained by sociodemographic or clinical variables. Then, it is possible that a latent variable could account for this trend. From the standpoint of neuropsychology, a possible explanation for this might be the existence of post-TBI neuroendocrine changes. Previous research indicating the existence of a link between testosterone and sociosexuality could represent a basis for such modifications [10]; the effects of neuroendocrine post-TBI dysfunction on testosterone levels and its precursors could modify sociosexuality levels. In fact, posttraumatic hypopituitarism is an underdiagnosed complication of TBI [49] and reports indicating that TBI is a common cause of pituitary dysfunction are compelling [50–98]. The main gonadal male hormone is testosterone, which is essential for the development of secondary sexual characteristics and behavioral patterns [99]. In addition, evidence from animal models of sexuality following TBI indicates that TBI-induced hypopituitarism in male rats causes decreased testosterone production and changes in sexual behavior [100]. However, this interpretation must be considered with caution since we did not measure testosterone levels in our study participants. Hence, further research in individuals with TBIs of different severities needs to be conducted to determine if this is an actual contributing cause.

As expected and consistent with previous reports, our results showed that there are statistically significant differences according to sex in sociosexuality. The results of the current study support our hypothesis and add new evidence to the fact that, compared to women, men have higher levels of sociosexuality across cultures [9]. These findings corroborate a great deal of the previous work in the field of sociosexuality [8, 11, 14, 15, 37, 101–104]. The results are also in the same direction of Canadian reports of sexual attitudes and behaviors. Specifically, the results of a Canadian study revealed that, compared to women, men had more frequent sexual thoughts, were more likely to report having engaged in oral sex, had a lower age at first intercourse, had more sexual partners, and were more willing to have casual sex [105].

Theories from evolutionary and comparative psychology bring elements to try to understand the fact that, on average, men are more willing than women to engage in casual sex, as can be explained by the theory of parental investment and sexual selection. The literature in the area of evolutionary psychology suggests that, compared to males, viviparity and the development of the placenta placed an important burden of time and energy in females [106]. This differential investment would be responsible for hypothalamic distinctions in the course of evolution, with differential hormonal effects during the development of the brain. It is therefore likely that post-TBI neuroendocrine dysfunction could change the expression of these evolutionary characteristics. However, this interpretation needs to be considered with caution not only because we did not measure hormonal changes, but also because human sexual behavior does not rely only on hormones. Human sexuality is multifactorial and based on psychological traits, behaviors, and cultural specificities, among others. Studies incorporating a more environmental/sociocultural perspective in this area are thus warranted considering the complexity and inherent multidisciplinary nature of sexuality.

Our third main finding is that infidelity levels, with infidelity defined as a person being unfaithful in a committed monogamous relationship, were comparable in healthy controls and individuals with TBI. Also, there were no differences according to sex. Taken together, these results are the first to reveal the nature of attitudes toward infidelity following TBI. It can thus be suggested that attitudes towards infidelity following TBI are not different from those of healthy controls. Therefore, a possible explanation is that, after a TBI, people's attitudes toward infidelity do not change.

In contrast to earlier findings showing that an unrestricted sociosexual orientation is associated with a greater willingness to engage in infidelity [13], the results of the current study could not find evidence of this link. A possible explanation of this might be that we used a general infidelity scale, while Mattingly et al.'s study included ambiguous, deceptive, and explicit infidelity [13]. This lack of uniformity in instruments to measure infidelity is one of the challenges regarding research in this area and may be responsible for incongruent findings [2].

Surprisingly, infidelity scores were negatively associated with sociosexual behavior, sociosexual attitudes, and sociosexual desire in individuals with TBI but not in healthy controls. This finding was unexpected and suggests that individuals with TBI reporting low levels in infidelity scores (e.g., disapproving of infidelity) also show high levels of SO (unrestricted SO). This finding indicates that individuals with TBI who express less acceptance of infidelity also report a more promiscuous mating strategy in terms of behavior (e.g., number of sexual partners in the last year), attitudes (e.g., imagining themselves enjoying casual sex with different partners), and desire (e.g., reporting a high frequency of spontaneous sexual fantasies with someone they have just met).

There are several possible explanations for these results. Firstly, individuals with TBI may have problems with emotional regulation that contribute to difficulties to control their own behavior [107]. As a consequence, thinking that being unfaithful in a committed monogamous relationship is not acceptable does not necessarily translate into regulating their own behaviors, attitudes, and desires regarding their willingness to engage in uncommitted sex. In fact, difficulties with behavioral regulation and social cognition are also common symptoms following TBI [108]. Another possible explanation is related to difficulties involving lack of awareness or anosognosia [109]. Anosognosia can affect emotional recognition and the interpretation of social signals [110]. This could explain the existence of this discrepancy where individuals with TBI can have difficulties in integrating what they do with what they think and what they feel. Another possible explanation to address this result can be related to hypersexuality. However, in a multicenter study, the estimated prevalence of inappropriate sexual behaviors following TBI was 8.9% and particularly evidenced in a minority of younger individuals with more severe injuries [111]. Such an explanation seems to be less probable as the sample of this study included a majority of milder injuries.

*Limitations and Future Directions*. The current study investigated the relationship between sociosexuality and attitudes towards infidelity following TBI. However, the results should be interpreted with caution in the face of several limitations. First, contrary to the epidemiological data of TBI in Canada, the sample included predominantly women with TBI whilst, regardless of age group, the overall rate of TBI is higher in men than women [112]. However, most of research conducted in sexuality and TBI has an underrepresentation of women [113]; so this could also be interpreted as one of the strengths of our study which included more than 45% of males. Furthermore, in the current study, 67% of TBI individuals had a mild TBI. Hence, caution is warranted in generalizing our results to moderate to severe TBI. In consequence, research on sociosexuality and infidelity needs to be conducted in larger samples, in particular with moderate to severe TBI.

Secondly, participants completed self-report measures to describe their sexual behavior. As sex is typically a highly private activity, people can conceal their true sexual behavior in an interview because sometimes they feel intensely embarrassed and threatened and may experience fear of reprisals when asked to reveal their sexual life [114]. However, to increase the validity of self-reported sexual behavior and avoid self-presentation bias, the questionnaires were completed anonymously. The study was conducted in a province that is highly open with respect to sexuality. For example, the results of a study revealed that people living in Quebec were more likely than participants from all other regions of Canada to report an interest in engaging in casual sex [105]. In this respect, our results cannot be extrapolated to other countries with different cultural backgrounds, especially those with more conservative attitudes towards sexuality. Future research should therefore concentrate on the investigation of cultural differences in sociosexuality and attitudes towards infidelity, by carefully controlling for methodological difficulties, such as presentation bias, among others [115].

As a third limitation, the current study was correlational/cross-sectional; so it was not possible to infer directional relationships between sociosexuality and attitudes towards infidelity in this group of individuals with TBI. Consequently, we cannot make inferences about causation and our interpretations should be treated as exploratory hypotheses. Prospective and longitudinal studies with larger samples will allow further more solid study of attitudes towards infidelity. The reasons are twofold: attitudes can change over time and also the relationships between attitudes with other psychological variables can also change with time. More broadly, additional research is required to understand this dynamics. This information would be useful when addressing psychosexual issues in individuals with TBI.

Finally, our study included exclusively a small sample of adults and, as such, results cannot be generalized to teenagers or older adults with TBI. In fact, most research has focused on adult TBI brain-behavior correlates with minimal involvement of adolescents [116]. In consequence, research needs to examine adolescents with TBI regarding sociosexuality and attitudes toward infidelity. Also, it would be interesting to compare experiences of sexually diverse people regarding infidelity and sociosexuality, such as lesbian, gay, bisexual, transgender, and intersex individuals with TBI [117].

As a closing remark, little attention has been given to within-sex individual differences in the type of infidelity found to be more distressing. This was not part of our study objectives. However, it is recommended to explore the hypothesis that greater sexual permissiveness (e.g., higher scores on sociosexuality) is associated with greater distress to sexual infidelity [4]. Sexual and emotional types of infidelity need to be addressed following TBI.

Despite all these limitations, the present study makes a unique contribution to the field of sexuality following TBI. Our study provides additional evidence with respect to sociosexuality after TBI and suggests a possible link between evolutionary psychology and neuropsychological effects of TBI. A better understanding of the interplay between biological, psychological, and sociocultural processes is needed to do justice to the complexity of this subject matter [118], in particular as it is expressed or modified following TBI.

## 5. Conclusions

This paper reports the fact that men with TBI show a trend towards the reduction of sociosexuality levels, suggesting the possible modification of a complex and deeply rooted psychosexual trait after a TBI. In addition, our results confirm that there are statistically significant differences according to sex in sociosexuality, supporting previous evidence that, compared to women, men have higher levels of sociosexuality across cultures. Finally, our findings indicate that although infidelity was comparable in healthy controls and individuals with TBI, individuals with TBI who express less acceptance of infidelity also report a more promiscuous mating strategy in terms of behavior, attitudes, and desire. This work contributes to existing knowledge in the field of sexuality and psychosexual changes following TBI. Taken together, the main theoretical implications correspond to the development of a link between evolutionary psychology and neuropsychology.

## Figures and Tables

**Table 1:** Sociodemographic characteristics of the TBI and healthy control samples (*N* = 89).

	TBI	Healthy controls	TBI	Healthy controls
	Frequency (%)		Mean (SD)	
Gender				
Male	19 (45.2%)	24 (51.1%)		
Female	23 (54.8%)	23 (48.9%)		
Race and ethnicity				
White	38 (90.5%)	45 (95.7%)		
Hispanic	4 (9.5%)	2 (4.3%)		
Work status				
Full time	16 (38.1%)	26 (55.3%)		
Part time	7 (16.7%)	7 (14.9%)		
Unemployed	19 (45.2%)	13 (27.7%)		
Missing	0 (0%)	1 (2.1%)		
Relationship status				
Single	26 (61.9%)	21 (44.7%)		
Married	4 (9.5%)	5 (10.6%)		
Separated	0 (0%)	4 (8.5%)		
Divorced	2 (4.8%)	2 (4.3%)		
Common-law	10 (23.8%)	14 (29.8%)		
Widow/widower	0 (0%)	1 (2.1%)		
Age (years)			37.9 (9.7)	37.6 (10.7)
Education (years)			12.8 (3.3)	13 (3.0)
Annual income (CAD)			39 007.5 (19 239.6)	31 975.6 (18 909.9)

*Note. *CAD, Canadian dollars.

**Table 2:** Clinical characteristics of the sample of individuals with TBI (*N* = 42).

	Frequency (%)	Mean (SD)
Cause of the injury		
Motor vehicle accident	18 (42.9%)	
Violence	2 (4.8%)	
Falls	4 (9.5%)	
Sports-related	6 (14.3%)	
Work accident	6 (14.3%)	
Other	3 (7.1%)	
Missing	3 (7.1%)	
LOC		
Yes	21 (50%)	
No	18 (42.9%)	
Missing	3 (7.1%)	
PTA		
Yes	20 (47.6%)	
No	19 (45.3%)	
Missing	3 (7.1%)	
Positive CAT or MRI		
Yes	25 (59.5%)	
No	10 (23.8%)	
Missing	7 (16.7%)	
Severity of the injury		
Mild TBI	28 (66.8%)	
Moderate TBI	3 (7.1%)	
Severe TBI	8 (19%)	
Missing	3 (7.1%)	
GCS		12.5 (3.6)
Years after injury		3.3 (4.3)
Length of LOC (hours)		5.8 (28.8)
Length of PTA (hours)		80.8 (203.8)

*Note. *LOC, loss of consciousness; PTA, posttraumatic amnesia; CAT, computed axial tomography; MRI, magnetic resonance imaging; and GCS, Glasgow coma scale.

**Table 3:** Means, standard deviations, and analysis of variance (ANOVA) results for sociosexuality and infidelity as a function of group and sex.

Measure	TBI		Healthy controls		ANOVA *F*		
	M	SD	M	SD	Group (G)	Sex (S)	G × S
SOI-R					1.0	7.2^*∗*^	0.6
Female	30.7	14.8	31.3	12.9			
Male	36.9	19.2	43.1	16.0			
SOI-BEH					0.3	2.5	3.5
Female	8.2	5.0	6.6	3.0			
Male	7.8	7.3	11.0	7.6			
SOI-ATT					2.0	3.3	0.1
Female	14.1	7.8	15.7	7.2			
Male	16.4	7.9	19.2	6.2			
SOI-DES					0.1	10.3^*∗*^	0.0
Female	8.3	4.9	9.0	5.5			
Male	12.7	7.9	12.9	5.9			
ATIS					0.7	0.6	0.0
Female	62.7	12.9	60.5	15.4			
Male	60.7	14.8	58.0	10.9			

*Note*. ^*∗*^ *p* < 0.05.

SOI-R, total score of the Sociosexual Orientation Inventory-Revised; SOI-BEH, sociosexual behavior; SOI-ATT, sociosexual attitudes; SOI-DES, sociosexual desire; and ATIS, Attitudes toward Infidelity Scale.

**Table 4:** Correlation matrix between infidelity, sociosexuality, and brain injury characteristics.

		1	2	3	4	5	6	7
1	ATIS	—						
2	SOI-R	−0.58^*∗∗*^	—					
3	SOI-BEH	−0.34^*∗*^	0.77^*∗∗*^	—				
4	SOI-ATT	−0.57^*∗∗*^	0.84^*∗∗*^	0.45^*∗∗*^	—			
5	SOI-DES	−0.49^*∗∗*^	0.84^*∗∗*^	0.52^*∗∗*^	0.57^*∗∗*^	—		
6	GCS	0.12	−0.30	−0.22	−0.30	−0.21	—	
7	Years after TBI	0.17	−0.18	−0.15	−0.27	0.01	0.05	—
8	PTA (hours)	−0.01	0.18	0.31	0.12	0.02	−0.58^*∗∗*^	−0.05

*Note*. ^*∗*^ *p* < 0.05; ^*∗∗*^ *p* < 0.01.

ATIS, scores of the attitudes toward infidelity scale; SOI-R, total score of the Sociosexual Orientation Inventory-Revised; SOI-BEH, sociosexual behavior; SOI-ATT, sociosexual attitudes; SOI-DES, sociosexual desire; GCS, Glasgow coma scale; and PTA, posttraumatic amnesia.
